# Criticizing Danaher’s Approach to Superficial State Deception

**DOI:** 10.1007/s11948-023-00452-2

**Published:** 2023-08-17

**Authors:** Maciej Musiał

**Affiliations:** Faculty of Philosophy, Adam Mickiewicz University in Poznań, Poznań, Poland

**Keywords:** Robots, Moral status, Ethical behaviourism, Deception

## Abstract

If existing or future robots appear to have some capacity, state or property, how can we determine whether they truly have it or whether we are deceived into believing so? John Danaher addresses this question by formulating his approach to what he refers to as superficial state deception (SSD) from the perspective of his theory termed ethical behaviourism (EB), which was initially designed to determine the moral status of robots. In summary, Danaher believes that focusing on behaviour is sufficient to determine whether SSD occurs. My general claim is that Danaher’s approach to SSD based on EB is implausible since it results in the impossibility of conceptualizing SSD, e.g., it does not enable determining whether or not SSD occurs in a particular case. Moreover, I show how Danaher’s approach to SSD needs to be transformed to become plausible. To make my point, I (1) examine the main features of EB and distinguish its two versions by showing how Danaher revised the original EB in response to criticism; (2) discuss Danaher’s approach to the problem of deception from the perspective of EB; (3) criticize that approach by showing that it requires revisions analogous to those that have already been recommended in reference to EB, and (4) propose an alternative method for determining the presence of SSD that covers diverse, plausible approaches to SSD.

## Introduction

One of the most frequently discussed issues regarding robots is the problem of deception, which is expressed by the following question: do robots truly possess the properties, capacities and states that they seem to possess, and how can we determine it? Most generally, deception occurs when we consider robots to possess some properties, capacities and states that they appear to but do not actually possess. Frequently, robotic deception is considered to result in various negative consequences and is thought to be wrong in and of itself (Sparrow, 2002; Sparrow & Sparrow, 2006; Turkle, 2007, 2010; Wallach & Allen, 2009; Sharkey & Sharkey, 2012; Sharkey & Sharkey, 2020). However, some approaches claim that although deception is generally harmful and has negative consequences, it can be acceptable if it serves a greater good (Isaac & Bridewell, 2017). Finally, some accounts suggest that the problem with deception results from some philosophical ideas, such as the endorsement of dualism between reality and appearance, that are unclear and raise many doubts; therefore, the very concept of deception should be deeply reconsidered and reformulated (Coeckelbergh, 2018).

John Danaher approaches the problem of deception by distinguishing its three major types (Danaher, 2020b). These types include superficial state deception (SSD), which is probably the most frequently discussed type of robotic deception. As its name suggests, SSD occurs when a robot appears to possess some states (or capacities and properties) but does not. Danaher approaches SSD from the perspective of ethical behaviourism (EB), originally introduced in reference to the problem of the moral status of robots.

My general claim is that Danaher’s approach to SSD based on EB is implausible. In particular, I show that following Danaher’s main assumption that behaviour is sufficient to determine whether SSD takes place results in the impossibility of conceptualizing SSD. I also show how Danaher’s approach to SSD needs to be transformed. In doing so, I draw from a critique that has already been made regarding EB. However, what differentiates my approach from the previous criticisms is (1) applying the critique to Danaher’s approach to deception, not to the problem of moral status, and (2) proposing an alternative approach to SSD.

To justify the abovementioned claims, I will (1) examine the main features of EB and distinguish its two versions by showing how Danaher revised the original EB in response to criticism; (2) discuss Danaher’s approach to the problem of deception from the perspective of EB; (3) criticize that approach by showing that it requires revisions that are in many respects analogous to those that have been incorporated into EB itself; and (4) propose an alternative method for determining the presence of SSD that covers diverse, plausible approaches to SSD.

## The Main Points of Ethical Behaviourism

Danaher developed the main version of EB in an article directly devoted to the presentation and defense of ethical behaviourism (2020a) – I will call this version The Original EB. However, after receiving some critical commentary, Danaher decided to modify The Original EB significantly by broadening both the relevant evidence and the acceptable ways of interpreting that evidence (2021) – I will call this version The Revised EB.

As mentioned, both versions of EB exhibit certain common features. Four of these features appear crucial. First, Danaher considers behavioural performances to constitute sufficient evidence for making decisions regarding the moral status of robots and whether they are deceiving us. That is, according to Danaher, if we want to determine the moral status of a robot or decide whether the robot is deceiving us, we can rely solely on the robot’s behavioural performance. Second, both versions of Danaher’s EB focus on analogy as a means of proceeding from evidence to conclusion concerning moral status or deceptiveness. Therefore, if we have already observed the behavioural performances of the robot in question, we should compare it with the behavioural performances of other entities, i.e., entities that possess moral status or other features that we want to identify regarding robots. Third, it is important to remain as neutral as possible regarding what Danaher most frequently labels “metaphysical properties”, “ontological grounding” or – following Sven Nyholm and Lily Frank (2017) – “what’s going on ‘on the inside’”. Hence, Danaher believes that when assessing robots’ moral status and deceptiveness, we should not wonder whether the robot has properties, states and capacities, such as consciousness, awareness, and emotions (since they cannot be accessed directly), but should focus on behavioural performance. Fourth, although Danaher encourages us to remain neutral toward what’s going on on the inside, he does not claim that properties such as consciousness or emotions do not exist, merely that they are not directly accessible – in this sense, Danaher embraces methodological behaviourism rather than ontological behaviourism. These four features are common to all three versions of EB. However, these versions exhibit relevant differences regarding their more specific characteristics, as shown below.

### The Original Ethical Behaviourism

Starting with The Original EB, Danaher characterizes its foundations in the following way: “If a robot is roughly performatively equivalent to another entity whom, it is widely agreed, has significant moral status, then it is right and proper to afford the robot that same status” (Danaher, 2020a, p. 2025). This claim is repeated more clearly and formally in The Comparative Principle of EB, which seems to express the core of this version of EB (and of other versions to some degree): “If an entity X displays or exhibits roughly equivalent behavioural patterns (P_1_…P_n_) to entity Y, and if it is believed that those patterns ground or justify our ascription of rights and duties to entity Y, then either (a) the same rights and duties must be ascribed to X or (b) the use of P_1_…P_n_ to ground our ethical duties to Y must be reevaluated” (Danaher, 2020a, p. 2030). Both of these statements are founded on and express the common features of EBs discussed above. The performative equivalence is the similarity between the behavioural performances of an entity that is a candidate for moral status and those of an entity that already has a moral status; thus, the behaviour is the crucial evidence that must be accounted for. However, it is worth noting that Danaher understands behaviour rather broadly in this context; he emphasizes the fact that when he refers to behavioural performances, he is referring not only to external physical behaviour but also to “internal” behaviour, e.g., observable brain behaviour (2020a, p. 2028). The progression from evidence to a conclusion requires the determination of similarities between the behavioural performances of both entities by engaging in analogical reasoning. Both formulations are neutral regarding what’s going on on the inside, and both avoid considering metaphysical properties; however, neither formulation claims that such properties do not exist.

### The Revised Ethical Behaviourism – Criticism of the Original Ethical Behaviourism and Danaher’s Response

As already mentioned, The Original EB has met some insightful criticism. Danaher devoted a separate paper to respond to some of the most important critical voices in this context, e.g., Smids (2020), Nyholm (2020) and Shevlin (2021), and significantly transformed EB as a result. My point in this section is not to examine the entirety of the criticism of EB and all of Danaher’s responses but rather to focus on two critical arguments that will be referenced by my criticism of Danaher’s approach to the problem of SSD. The first such argument pertains to the necessity of using abductive reasoning instead of analogical reasoning, and I will discuss the manner in which it is presented by Smids. The second argument refers to the relevance of what’s going on on the inside, and I will follow Nyholms’ approach to this issue (although, Smids discusses it as well).

With respect to the first argument, Smids (2020, pp. 2584–2588) claims that in daily practice, we do not limit ourselves to making analogies of observable behavioural performances. In his opinion, instead of analogical reasoning, we engage in abductive reasoning and make inferences from behavioural performances to arrive at the best explanation of such performances. Therefore, according to Smids, “the strongest version of EB” – the version that is coherent with actual daily practice and takes into account all the relevant data – should rely not on the behaviour itself and comparisons among analogical behavioural performances but rather on the inferences that can be made from this behaviour to obtain the best explanation for it. It can be said that the fundamental aspect of this argument is to focus not merely on how the particular entity behaves but also on the reason it behaves in such a way. Danaher partially agrees with Smids. Instead of admitting that analogical reasoning is insufficient and that EB requires analogical reasoning, he claims that both of these reasonings can be applied to EB: “you can derive an argument from analogy that makes the case for a particular being’s moral standing (…) but you can also derive other kinds of argument, such as an inference to best explanation” (2021, p. 474). This shift is significant, since in The Original EB, The Comparative Principle of EB unambiguously identifies analogy as the proper way of proceeding from behavioural evidence to conclusions regarding moral status. Thus, The Revised EB embraces abduction as a valid way of proceeding from evidence to the conclusion (although it does not endorse the claim that analogy is invalid or insufficient in that regard).

Regarding the second argument, Nyholm (2020, pp. 116–119) criticizes Danaher’s application of ethical behaviourism to the problem of robotic friendship to argue that what’s going on on the inside matters and cannot be reduced to or replaced by behaviour. Nyholm claims that behaviour provides us with epistemic reasons to believe that someone is our friend (or, more generally, that the person in question possesses some capacity, property or state) but that the axiological reasons for our tendency to value someone as a friend do not rely on behaviour but rather on what’s going on on the inside of that entity. Hence, Nyholm asserts that even when behavioural performances do not differ, we may evaluate them differently depending on whether we believe they express certain actual properties, states and capacities. Danaher responds to Nyholm by agreeing that what’s going on on the inside matters but adds that behaviour is ultimately the only evidence that can identify whether any internal capacities, states or properties are present. While Danaher made such declarations even in his formulation of The Original EB, the response to Nyholm seems to represent a relevant shift since Danaher no longer claims that “what’s going on ‘on the inside’ *does not matter* from an ethical perspective” (2020a, p. 2025) but admits that he “cannot be strictly neutral with respect to the ontological grounding” (2021, p. 474). Hence, The Revised EB acknowledges that what’s going on on the inside matters.

However, although Danaher partially agrees that abductive reasoning may be useful and that what’s going on on the inside may matter, he still maintains that behaviour is sufficient evidence for ascribing moral status and that analogical reasoning is a proper way to achieve that aim: “no matter which precise property or set of properties is thought to ground moral standing, behavioural equivalence will always provide evidence for its presence. Alternatively, and more boldly, you can argue that even if there is some doubt as to the exact set of mental properties that grounds moral status, behavioural equivalence of a diverse and consistent type should be enough to convince you that an entity possesses moral status”. (“I do not know exactly what it is that grounds moral status but this thing sure looks and acts consistently like other beings that have moral status so it probably does too”)” (2021, p. 474). In other words, Danaher seems to believe that The Comparative Principle of EB remains plausible. I will show that maintaining such an approach to the problem of superficial state deception is implausible and results in an inability to conceptualize this kind of deception at all. Furthermore, I will demonstrate how to revise Danaher’s approach to plausibly account for SSD.

## Superficial State Deception from the Perspective of Ethical Behaviourism

In his initial definition of deception, Danaher asserts that “deception arises whenever signals or representations are used to create a false or misleading impression” (Danaher, 2020b, p. 120). However, he also distinguishes the three following types of deception that pertain to robots: “external state deception – the robot uses a deceptive signal regarding some state of affairs in the world external to the robot”; “superficial state deception – the robot uses a deceptive signal to suggest that it has some capacity or internal state that it actually lacks”; and “hidden state deception – the robot uses a deceptive signal to conceal or obscure the presence of some capacity or internal state that it actually has” (Danaher, 2020b, p. 120). In the following, I focus only on superficial state deception.

It is worth clarifying whether Danaher speaks about deception on the metaphysical (does SSD exist in robots?), epistemological (how can we know whether SSD takes place?), or ethical (is SSD in robots right or wrong?) level. First, I believe that since Danaher constantly emphasizes that EB does not make any metaphysical claims, his approach to SSD from the perspective of EB is not on the metaphysical level. Second, while Danaher consequently introduces EB – also by its very name – as an ethical theory, he presents no considerations on whether SSD is ethically right or wrong (in fact, he claims that EB aims to reduce the ethical problem to an epistemological one (2020a, footnote 6)). Hence, I assert that what Danaher offers are epistemological investigations about the issue of determining whether SSD occurs or not and I also limit myself to this level of consideration.

Danaher explicitly proposes to perceive SSD from the perspective of EB: “superficial state deception is best interpreted through the lens of a philosophical theory—here called ‘ethical behaviourism’” (Danaher, 2020b, p. 117). One may wonder how a theory designed to determine the moral status of robots can be applied to the problem of deception. After all, moral status and deception are separate issues. However, both moral status and SSD pertain – at least according to most approaches – to the problem of what’s going on on the inside. That is, both issues focus on identifying the genuineness of some appearing phenomena by investigating the presence of some (internal) states, properties or capacities that could justify the abovementioned genuineness. However, Danaher asserts that the application of EB to SSD (similarly as in the case of moral status) “significantly alters how we ought to think about it” (Danaher, 2020b, p. 117). This alteration most generally equals the belief that behaviour is sufficient to determine whether SSD occurs; thus, considering what’s going on on the inside, ontology or the designer’s intentions is not necessary. Now, I will examine in more detail how exactly the presence (or absence) of SSD should be determined according to Danaher’s approach.

Danaher consequently claims that the criteria for assessing deceptiveness are consistency and completeness of behavioural performances: “the genuineness of a capacity or mental state depends on both the richness of the set of superficial states from which you infer its presence (its *completeness*) and its *consistency*” (2020b, p. 124, italics in original). Hence, to determine whether superficial signals (e.g., behavioural performances) are deceptive or genuine, one must check whether these signals are complete and consistent.

Moreover, while Danaher does not state this very clearly, he seems to suggest that to assess the consistency and completeness of behavioural performances of a robot, one should compare them with behavioural performances of a being whose behavioural performances are considered genuine. In particular, when he speaks about “a robot that signals some forms of affection, but does not perform all the acts of care and affection that we usually associate with human friends and companions” (2020b, p. 124), he suggests comparing robot and human behavioural performances. This approach seems reasonable since Danaher’s EB is generally based on comparisons and analogies and because without such a point of reference, it would not be known how to decide what level of completeness and consistency and in what exact respects should take place for behaviour to be considered genuine (yet, I will show further on, that this problem is not entirely solved by making analogies). Hence, it seems that in applying EB to SSD, it is also possible to adapt The Comparative Principle of EB. I believe it can be renamed “The Comparative Principle of SSD” and formulated in the following way:

If an entity X displays or exhibits behavioural patterns (P_1_…P_n_) that are roughly equivalent to behavioural patterns (P_1’_…P_n’_) displayed or exhibited by an entity Y in terms of consistency and completeness and if it is believed that those patterns are not superficially deceptive in the case of Y, then either (a) the same genuineness must be ascribed to behavioural performances of X or (b) the genuineness of P_1_…P_n_ in the case of Y must be reevaluated.

### Superficial State Deception Cannot Be Conceptualized by Ethical Behaviourism

The Comparative Principle of SSD is a very general statement, and to be put into actual practice, it requires some “calibrations” when applied to particular cases. For instance, it is necessary to know how to determine which behavioural performances should be considered when making comparisons and to what degree and in what respect their completeness and consistency should be equivalent. In fact, a similar problem appears in the case of using EB and The Comparative Principle of EB to assess the moral status of robots. Danaher’s general answer to the first question in reference to moral status ascription focuses on cognitive behaviour, claiming that EB “is the ethical equivalent of the Turing Test” (2020a, p. 2027). For the second question, he refrains from delivering any decisive answer (apart from the suggestion that the performative threshold should be lower than higher, since “we should err on the side of over-inclusivity” (2020a, p. 2023). In this section, I will demonstrate that there is no way to answer the two abovementioned questions plausibly and put the Comparative Principle of SSD into practice by referring only to behavioural performances and analogies between them. In other words, I argue that ethical behaviourism in general and The Comparative Principle of SSD in particular cannot conceptualize superficial state deception. Moreover, I show the modifications required to enable proper conceptualization.

### Analogous Behaviour Is Not Sufficient. The Same Behaviours Can Mean Different Things

I would like to make my point by referring to the example of determining the deceptiveness of behaviour that suggests love (I choose that particular topic partially because Danaher also briefly refers to it).

Scenario 1: Person A thinks that person B’s behaviour is deceptive in terms of being an expression of love because B does not want to marry A. Even though B exhibits all the behaviour that is generally considered an expression of love in the culture to which A and B belong, and all the available data – including lie detectors and fMRI findings – confirms that B’s behaviour genuinely expresses love, A asserts that B’s behaviour is deceptive. Now, let us consider two following alternative scenarios.

Scenario 2: B decides to marry A. After getting married, B’s behaviour is far less complete and consistent in being an expression of love. However, A believes that B’s love is not superficially deceptive.

Scenario 3: B does not decide to marry A. As a result, A breaks up with B, and B forms a new relationship with C. B’s behaviour toward C is identical to their behaviour toward B, including the unwillingness to marry C. C does not find B’s behaviour deceptive but a genuine expression of love.

The first and second scenarios demonstrate that some behavioural performances might be far more important for assessing the deceptiveness of love than others. However, this importance does not originate from the behaviour itself but from the inferences we make from this behaviour, e.g., the best explanations of that behaviour. Only if we decide what particular behavioural performance means can we decide whether – and to what degree – it is relevant for assessing deceptiveness. In this sense, behaviour and its comparison are insufficient for assessing superficial state deception: inferences must be made from the behaviour to decide which behaviour is relevant and to what degree and in what respects – otherwise, we cannot decide what and how matters.

The third scenario, when compared with the second scenario, demonstrates that the same behaviour – also in terms of its completeness and consistency – can be regarded as either superficially deceptive or genuine. Of course, one could argue that only one of these approaches is right. However, my point is that regardless of whether we would like to decide which of these approaches is correct or assume that both of them are, what grounds the decision is not solely the behaviour itself but also the inferences made from the behavioural performances and their best explanations. Again, maybe only one of these inferences and explanations is correct. However, even if there is only one correct inference and explanation for each particular behaviour, then this inference and explanation, not solely the behaviour itself, enables us to make a decision about which behaviours are relevant in assessing whether behavioural pattern is superficially deceptive or not and whether this behaviour is sufficiently equivalent in terms of completeness and consistency and, eventually, deceptive or genuine.

In summary, what matters are the inferences we make from behavioural performances and their best explanations. These explanations enable us to check whether behavioural performances are complete and consistent and to determine their superficial deceptiveness. Otherwise, the “raw” behaviour itself is meaningless unless some inferences are made from it and it is explained or interpreted.

To express my point otherwise, I would like to note that when Danaher says that “Superficial states can be incomplete or inconsistent, but not directly deceptive in and of themselves” (2020b, p. 122), he is right in the second part of the sentence but should add that superficial states also cannot be complete or consistent in and of themselves. Similarly, when Danaher claims in his discussion with Shevlin that “cognitive architectures do not speak for themselves. They speak through behaviour” (Danaher, 2021, p. 476), I believe he is right only in the former claim, while he is wrong in the latter one. Behaviour does not speak for cognitive architectures or even for itself. Both cognitive architectures and behavioural performances do not speak – and do not introduce themselves as consistent or inconsistent, complete or incomplete, deceptive or genuine – until *we* speak: make inferences from them and explain them. Only inferring and explaining enable us to make sense of them and meaningfully compare them with another behaviour, particularly by deciding what exactly should be compared and to what degree similarities should occur. Hence, behaviour and analogy themselves are insufficient to assess whether some behavioural performances and patterns are superficially deceptive. Instead, it is required to make inferences from the behaviour to achieve the best explanation of a particular behavioural performance and to compare behavioural performances and their explanations. As a result, The Comparative Principle of SSD must be modified in the following way:

If an entity X displays or exhibits behavioural patterns (P_1_…P_n_) that are roughly equivalent to behavioural patterns displayed or exhibited by an entity Y in terms of consistency and completeness and in terms of their best explanations, and if it is believed that those patterns are not superficially deceptive in the case of Y, then either (a) the same genuineness must be ascribed to behavioural performances of X or (b) the genuineness of P_1_…P_n_ in the case of Y must be reevaluated.

### Analogous Behaviour Is Not Necessary. Different Behaviours Can Mean the Same Thing

In the version of The Comparative Principle of SSD presented above, behaviour and analogy are accompanied by inferences and abduction. As a result, behavioural patterns and inferences made from them should be roughly equivalent. However, it is worth noting that it is not necessary for behavioural performances to be equivalent since the equivalence of the inferences and best explanations is sufficient. In other words, we can assess some behavioural performance as genuine or superficially deceptive solely on the basis of comparing its inferences and the best explanation with inferences and the best explanations of behavioural performances of others that we do not find deceptive. Resigning from the condition of equivalence of behaviour enables the principle to be applied to a wider range of cases and helps to avoid some problems.

To understand the above, it is worth noting that very diverse and disanalogous behavioural patterns can be equally genuine in reference to the very same internal state. For instance, there are various ways of expressing love in various human societies and cultures. There is even more variety in behavioural expressions of diverse emotions when we consider nonhuman animals. Some have more differences than similarities, and it seems that the behavioural patterns of robots in love might also be disanalogous to those of human beings. However, one could claim that all these differences refer mostly to the “external” behaviour, while the “internal” behaviour (e.g., behaviour of the brain and other internal organs) remains analogous. To answer that, it is worth recalling that Danaher maintains that robots’ ontology – including what he and Shevlin label “cognitive architecture” – does not have to be analogous to humans’ (or animals’) ontology either with respect to the material they are built or their structure. Therefore, if Danaher allows robots to be built and structured differently than humans and animals, how does he like to compare their behaviours, especially “internal” ones? How can we compare the behaviour of a brain with the behaviour of something that is disanalogous from a brain both in its substance and structure, and how can we decide whether their completeness and consistency are roughly equivalent?^1^ There might be some way to do that, but Danaher does not demonstrate it. However, I believe that even if such a way exists, it is unnecessary to use it.

My point is that comparing behavioural performances is not only problematic, but also unnecessary since comparing the best explanations of the behavioural patterns is sufficient. After all, if we build a robot that behaves, both externally and internally, utterly differently from any other entity we know, and our best explanation of that behaviour would be that this robot is, for instance, genuinely in love, should we resign from ascribing capacity for love to the robot only because it exhibits disanalogous behaviour? I suppose that the most common answer would be “no”, and I assert that Danaher would also embrace this answer since he advocates erring “on the side of over-inclusivity” (2021a, p. 2023) (moreover, to be clear, Danaher also argues that “ethical behaviourism is (…) not a claim about the kinds of evidence that are necessary for moral standing” (2021, p. 474), which means that he would probably agree with the claim expressed in the first part of a title of this section). Hence, if comparing behaviour is not only highly problematic but also unnecessary, since what requires comparison are explanations of behaviour, The Comparative Principle of SSD requires further modifications:

If an entity X displays or exhibits behavioural patterns (P_1_…P_n_) that are roughly equivalent in terms of their best explanation to behavioural patterns (P_1’_…P_n’_) displayed or exhibited by an entity Y and if it is believed that patterns (P_1’_…P_n’_) are not superficially deceptive in the case of Y, then either (a) the same genuineness must be ascribed to behavioural performances (P_1_…P_n_) of X or (b) the genuineness of (P_1’_…P_n’_) in the case of Y must be reevaluated.

### Behaviour Is Not the Only Evidence and Abduction Is Not the Only Interpretation

I would like to make two more modifications to the principle. First, if behaviour is not sufficient and not even necessary, then there is no point in limiting the range of evidence only to behaviour. Hence, in determining whether behaviour is superficially deceptive, one might consider not only the behaviour itself but also all available evidence, e.g., designer intentions, the ontology of an entity in question or the subjective experience of one who relates to this entity in cases in which this or other evidence is found relevant. In summary, to achieve the best explanation of a robot’s behaviour, we should make inferences not only from the behaviour but also from all the available evidence.

Second, to make The Comparative Principle of SSD as general as possible, I would like to acknowledge that “the best explanation” should be understood as broadly as possible, not necessarily as a result of rigorously conducted abductive reasoning. Whatever kind of explanation and/or interpretation one considers the most plausible, it – or rather its results – can be incorporated into the principle. As a result, the final version of The Comparative Principle of SSD is as follows:

If an entity X displays or exhibits behavioural performances (P_1_…P_n_) that are roughly equivalent in terms of the best explanations and/or interpretations of them and all other relevant evidence to behavioural performances (P_1’_…P_n’_) displayed or exhibited by an entity Y, and if it is believed that behavioural performances (P_1’_…P_n’_) are not superficially deceptive in the case of Y, then either (a) the same genuineness must be ascribed to behavioural performances (P_1_…P_n_) of X or (b) the genuineness of (P_1’_…P_n’_) in the case of Y must be reevaluated.

## What is the Point of the Comparative Principle of Superficial State Deception?

I assert that The Comparative Principle of SSD provides the most general and broad way to determine the presence or absence of what Danaher labels superficial state deception. In short, it advocates making inferences from all the evidence considered relevant and comparing the achieved best explanation/interpretation of behaviour with the best explanations/interpretations of behaviours that we consider genuine. In other words, it emphasizes – in contrast to Danaher’s approach – that evidence itself is not enough to assess the presence of SSD since the interpretation of the evidence is necessary. To be clear, The Comparative Principle of EB remains neutral in ethical considerations about whether the presence or absence of SSD is good or bad. Instead, it focuses solely on the epistemological task of determining whether SSD occurs in a particular situation. Moreover, it does not offer any firm answer to that question by itself but rather provides an abstract frame that covers diverse approaches that may variously specify and clarify what is the relevant evidence, what is the best interpretation/explanation of that evidence, and what is relevant in comparing these interpretations/explanations.

I believe that The Comparative Principle of SSD is general and broad enough to cover most of the ways of thinking about superficial state deception that can conceptualize it. I cannot prove it here for all such approaches, yet I will briefly show that it is coherent with one of them: relational turn (RT). The relational turn has some common features with ethical behaviourism, as Danaher himself notes (2020a, pp. 2037–2039), and as some critics of both of these approaches do (Müller, 2021; Köhler, 2023). While it is impossible to give all the justice to RT here, I will try to demonstrate that The Comparative Principle of SSD is generally compatible with it.

### The Comparative Principle of Superficial State Deception and Relational Turn

RT was established by Gunkel (2012, 2018) and Coeckelbergh (2012; Coeckelbergh & Gunkel, 2014) and followed and/or advanced by Gellers (2020) and Jecker (2021; Jecker et al., 2022), among others. To repeat, there is no way to give justice to all the aspects of this approach here, not only because it is in many ways unconventional and subtle but also because there are some differences between the main representatives of this approach (for instance, in a recent article about anthropomorphism, which has much in common with superficial state deception, Coeckelberg (2022) builds his view in opposition to David Gunkel’s), and there are many interesting criticisms that are worth discussing while referring to this approach (Barrow, 2023; Gerdes 2022; Gamez, 2022; Birhane & van Dijk, 2020a; Birhane & Dijk, 2020b; Sætra 2021). I also cannot speak on the main topic of interest of this approach – moral status of robots and conditions of its ascription – since this paper is devoted to the issue of (superficial state) deception. Instead, I will try to apply the main ideas of RT to the issue of superficial state deception and refer to the direct approach to the problem of deception formulated on the grounds of RT. While this topic deserves a separate and more detailed approach, I will address it as briefly as possible here.

Generally, Gunkel and Coeckelbergh share Danaher’s skepticism regarding “what is going on on the inside”, but they take it a step further and claim that “what’s going on on the outside”, e.g., the observable properties such as behavioural performances, are also not the phenomenon we should focus on. In their opinion, properties are “a posteriori product of extrinsic social interactions” (Gunkel, 2018, p. 200) or – more succinctly – “relations are ‘prior’ to the relata” (Coeckelbergh, 2012, p. 45). In this way, I believe they want to emphasize that before we can determine anything meaningful about properties, we must relate with an entity that possesses them and interpret them. Moreover, proponents of RT emphasize that these interpretations depend on individual characteristics and various contexts, which entails that relations and interpretations are highly diverse. Acknowledging this diversity, representatives of RT emphasize that anthropocentric and Western-centric approaches are not the only and probably not even the best positions. Furthermore, to refer to one of the direct approaches to deception formulated on the grounds of RT, Coeckelbergh (2018) proposes to redescribe and reevaluate the issue of deception by replacing the conceptualization that is based on what he calls the Platonic distinction between reality and illusion by focusing on performances, narratives and experiences that occur within the actual relations with a robot (or other AI artifact).

I assert that The Comparative Principle of SSD is compatible and coherent with the general claims of RT and with more specific features of Coeckelbergh’s redescription and reevaluation of deception. First, it acknowledges that inferences made from the properties, e.g., interpretations and explanations, are what eventually matters, not the properties themselves. Second, it does not postulate only one type of “the best explanation/interpretation” and does not list “all the relevant evidence”. Thus, it is not necessarily Western-centric since it constitutes a general form that can embrace various kinds of relevant evidence and various kinds of interpretations: for instance, it can account for an interpretation of a relation with a particular entity (e.g., robot) and the subjective experience that accompanies this relation. Third, The Comparative Principle of SSD is not anthropocentric, at least in the sense that it does not say that the entity that is a “role-play model” must be a human being.

Hence, I believe that The Comparative Principle of SSD is an abstract scheme that is general and open enough to embrace most approaches to determining whether a signal is deceptive. As for the Cockelbergh’s approach to deception, The Comparative Principle of SSD accounts for all the relevant evidence, including narratives, performances and experiences that occur within or as a result of relation. Moreover, the principle does not suggest any particular way of making inferences from this evidence and can embrace whatever one considers the best explanation/interpretation. Therefore, as long as the proponents of RT do not advocate rejecting the whole concept that some phenomena might be deceptive (and to the best of my knowledge, they do not go that far) or make redescriptions similar to Danaher’s that make it impossible to conceptualize superficial state deception (this is also something I do not think they try to achieve), The Comparative Principle of SSD does not collide with their approach.

## Conclusion

In this paper, I have criticized Danaher’s approach to superficial state deception from the perspective of ethical behaviourism and demonstrated how this approach could be corrected. However, this paper can also be understood as a more general suggestion regarding the issues of deception in robotics and the moral status of robots, particularly if Danaher’s approach to these matters is also perceived as a representative of some broader tendency. I believe that Danaher’s ambitious attempt to reduce ethical issues to epistemological ones and to depend solely on knowledge of observable behavior represents a tendency to believe that it is possible to make objective discoveries of facts that will provide us with certain and sufficient knowledge of whether deception takes place or moral status can be ascribed.

The general suggestion of this paper is that there is no way to determine the presence of deception or to ascribe the moral status of robots by depending solely on the knowledge of facts. Even if Danaher (and many others) is right that we should set aside metaphysical properties (which is not clear), we cannot ignore that facts without interpretation do not mean anything and that the question “What are robots?” is actually always “What are robots for us?” By pointing out that assessing deception and ascribing moral status are inevitably matters of interpretation, I emphasize that they are not and should not be seen solely as a result of discovering certain facts about how the world really is and passively adjusting to this state of affairs but should also be seen as a consequence of actively, interpretively attributing meaning and making uncertain choices about values.

Obviously, values vary across times, cultures and individuals, so the same facts can mean different things, and different facts may mean the same thing. In this sense, I acknowledge the variety of approaches to deception and moral status, emphasize interpretation and meaning attribution as a source of this diversity and reject the view that this variety of interpretations can be set aside by discovering “raw” objective facts.

However, this paper does not address one of the crucial questions that might come to mind, namely, how to evaluate those various approaches. For instance, are they equally justified, or is it possible to show that only some of them are plausible? Answering these questions requires further work, and the insight that this paper provides is that this cannot be done solely on the level of epistemic considerations of discovering facts but should also be done on the level of ethical and axiological considerations regarding values we would like to achieve. Hence, this paper might be seen as suggesting that it is not enough to ask, “What are the *true* criteria for ascribing the moral status of robots and assessing the genuineness of their behavior?” Rather, we inevitably must ask, “What are *good* criteria for ascribing the moral status of robots and assessing the genuineness of their behavior?”

## Data Availability

Not applicable.

## Footnotes

1 In many approaches, these issues are considered intrinsically linked with issues from the field of philosophy of mind e.g. whether an entity that does not possess an organic brain but some inorganic hardware can possess awareness, consciousness, etc. While Danaher attempts to avoid these problems by focusing solely on behavior, there is an interesting critique that suggests that ethical behaviorism and a relational turn approach, can be found plausible only if they draw from some assumptions from the philosophy of mind debates (Köhler, 2023).
